# Raman Spectroscopy and Its Modifications Applied to Biological and Medical Research

**DOI:** 10.3390/cells11030386

**Published:** 2022-01-24

**Authors:** Elvin S. Allakhverdiev, Venera V. Khabatova, Bekzhan D. Kossalbayev, Elena V. Zadneprovskaya, Oleg V. Rodnenkov, Tamila V. Martynyuk, Georgy V. Maksimov, Saleh Alwasel, Tatsuya Tomo, Suleyman I. Allakhverdiev

**Affiliations:** 1 Russian National Medical Research Center of Cardiology, 3rd Cherepkovskaya St., 15A, 121552 Moscow, Russia; elvin21128@gmail.com (E.S.A.); rodnenkov@mail.ru (O.V.R.); trukhiniv@mail.ru (T.V.M.); 2 Biology Faculty, Lomonosov Moscow State University, Leninskie Gory 1/12, 119991 Moscow, Russia; gmaksimov@mail.ru; 3 K.A. Timiryazev Institute of Plant Physiology, RAS, Botanicheskaya str., 35, 127276 Moscow, Russia; venera.khabatova@ifr.moscow (V.V.K.); zadneprovskaya@ifr.moscow (E.V.Z.); 4 Geology and Oil-gas Business Institute Named after K. Turyssov, Satbayev University, Satpaeva, 22, Almaty 050043, Kazakhstan; kossalbayev.bekzhan@gmail.com; 5 Department of Biotechnology, Faculty of Biology and Biotechnology, Al-Farabi Kazakh National University, Al-Farabi Avenue 71, Almaty 050038, Kazakhstan; 6 Department of Physical Materials Science, Technological University “MISiS”, Leninskiy Prospekt 4, Office 626, 119049 Moscow, Russia; 7 Zoology Department, College of Science, King Saud University, Riyadh 12372, Saudi Arabia; salwasel@ksu.edu.sa; 8 Department of Biology, Faculty of Science, Tokyo University of Science, 1-3 Kagurazaka, Shinjuku-ku, Tokyo 162-8601, Japan; tomo@rs.tus.ac.jp; 9 Institute of Basic Biological Problems, RAS, Pushchino, 142290 Moscow, Russia

**Keywords:** carotenoids, lipid droplets, microalgae, Raman spectroscopy, Surface-enhanced Raman Spectroscopy

## Abstract

Nowadays, there is an interest in biomedical and nanobiotechnological studies, such as studies on carotenoids as antioxidants and studies on molecular markers for cardiovascular, endocrine, and oncological diseases. Moreover, interest in industrial production of microalgal biomass for biofuels and bioproducts has stimulated studies on microalgal physiology and mechanisms of synthesis and accumulation of valuable biomolecules in algal cells. Biomolecules such as neutral lipids and carotenoids are being actively explored by the biotechnology community. Raman spectroscopy (RS) has become an important tool for researchers to understand biological processes at the cellular level in medicine and biotechnology. This review provides a brief analysis of existing studies on the application of RS for investigation of biological, medical, analytical, photosynthetic, and algal research, particularly to understand how the technique can be used for lipids, carotenoids, and cellular research. First, the review article shows the main applications of the modified Raman spectroscopy in medicine and biotechnology. Research works in the field of medicine and biotechnology are analysed in terms of showing the common connections of some studies as caretenoids and lipids. Second, this article summarises some of the recent advances in Raman microspectroscopy applications in areas related to microalgal detection. Strategies based on Raman spectroscopy provide potential for biochemical-composition analysis and imaging of living microalgal cells, in situ and in vivo. Finally, current approaches used in the papers presented show the advantages, perspectives, and other essential specifics of the method applied to plants and other species/objects.

## 1. Introduction

In recent decades, Raman spectroscopy (RS) has been used in several studies on animal cells [1,2,3]. The method is popular among biophysicists and life science researchers. RS allows for the study of living cells in their natural conditions without any damage [4,5]. Nowadays, we can see a significant increase in the use of RS in plants, and especially in algae research.

RS is a well-known approach used in many of biomedical studies. Since biomolecules are involved, the main obstacle to the use of such methods in life science research is the low signal of Raman scattering. There are a number of modifications of RS that allow Raman scattering to be improved. There are existing approaches to detect Raman signals not only on the surface of human skin, but also inside the vasculature and various organs of patients. 

In this review, we have attempted to cover the list of modifications of RS applied to biological and medical research; moreover, algal research, and especially to understand more detailed mechanisms related to the biosynthesis and transport of lipid droplets/fatty acids and carotenoids. It is important to note that algal research might represent an interest in terms of carotenoids production and further application of carotenoids for medical treatment of human diseases.

**Figure 1:**
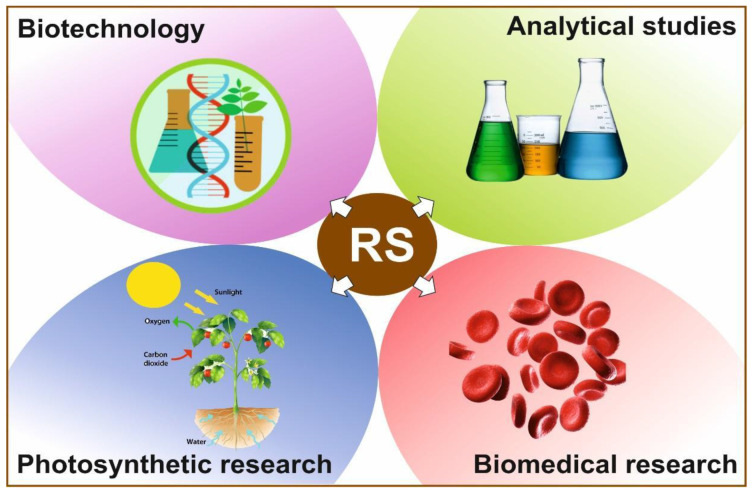
Application of RS in different research.

We have also covered studies on human cells [1,2] and microalgae [6,7] that we thought would be useful to introduce to the reader, especially in view of future studies of the use of algae. We have tried to cover the variety of algal species that have been used in different studies with the application of RS and its modifications. Figure 1 provides a schematic of the different research areas in which RS can be used. Biotechnological, biomedical, photosynthetic, and analytical research applications of RS are the main interest of this particular review.

## 2. The Principles of the Method of Raman Spectroscopy

**Figure 2:**
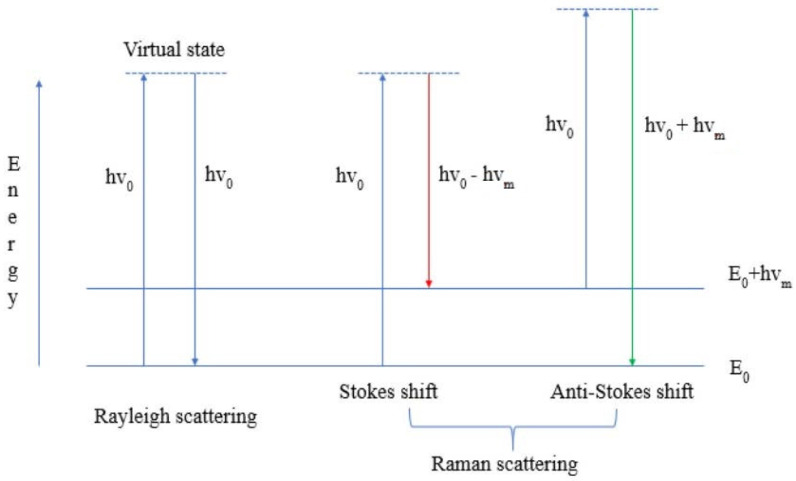
Energy transition of Rayleigh and Raman scattering.

Figure 2 shows the energy transition of Rayleigh and Raman scattering. The former (Rayleigh) is based on the principle that the frequency of the absorbed and scattered photon does not change—elastic scattering. In the latter (Raman), on the other hand, there is a shift in frequency of the scattered photon (change in energy or change in wavelength)—inelastic scattering. It is also essential to note that Raman scattering is divided into Stokes and anti-Stokes shifts. The Stokes shift is more likely because it is associated with the shift of the binding maxima to the longer wavelengths (the energy and frequency of the scattered photon are correspondingly lower than those of the absorbed photon—see Figure 2). The anti-Stokes shift, on the other hand, is less likely. It results from the shift of the binding maxima to the shorter wavelengths (energy and frequency of the scattered photon are correspondingly higher than those of the absorbed photon—see Figure 2).

In order to increase the sensitivity and resolution capability of the method, scientists use the approach based on the surface plasmon resonance effect. The electrons on the surface of the metal (silver and/or gold nanoparticles) oscillate. At a certain point, the frequency of the photon resonates with the frequency of the surface electrons. The surface plasmons substantially enhance the local electric field of the incident light (on the molecules near the surface’s vicinity of the metal nanoparticles) [8]. In recent decades, this effect has been used to modify the method of RS to apply it in biological and medical research.

## 3. Raman Spectroscopy and Its Modifications: Advantages and Use

**Figure 3:**
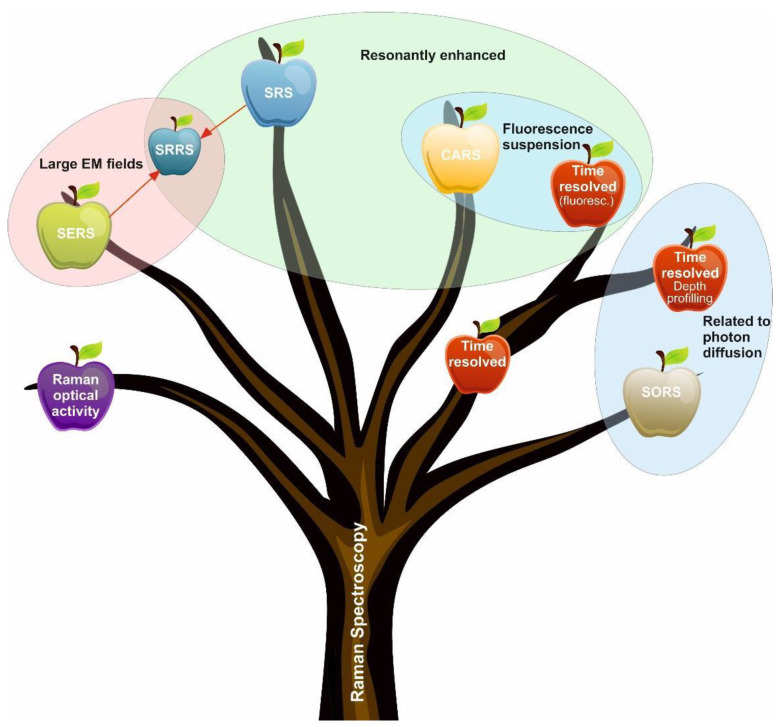
“Family tree” of the RS. The relatively simple RS is the root of the complex surface-enhanced, resonance-enhanced time—and spatially-resolved techniques. Abbreviations: SERS,  Surface-enhanced Raman Spectroscopy; CARS, coherent anti-Stokes Raman spectroscopy; RRS, resonance-enhanced Raman scattering; SORS, spatially offset Raman spectroscopy. Modified from Buckley and Ryder [9].

There are many variants of Raman spectroscopy, all of which use the phenomenon of Raman scattering in different ways (Figure 3). The choice of which variant to use for a particular measurement depends on inherent factors, such as the complexity of the sample and/or the concentrations of the target analyses.

The popularity of the RS method among biophysicists around the world is explained by a list of advantages of the method. RS is a non-invasive, rapid, and sensitive method for in vitro investigations [10,11,12]. Usually we face an obstacle—the biomolecules are present in the cell in very low concentrations, so the Raman scattering/signal is very low. To enhance the Raman scattering signal, the modifications of the method can be used (see Table 1), such as surface-enhanced Raman spectroscopy (SERS), coherent anti-Stokes Raman scattering (CARS), surface-enhanced Raman Spectroscopy (SERCS) [13], and micro-Raman spectroscopy [14].

As mentioned earlier, there is a special mechanism of Raman scattering (plasmon resonance) enhancement for modifications such as SERS. Moreover, each modification of the method can solve a specific task/objective.

The following sections describe the most commonly used types of RS.

### 3.1. Surface-Enhanced Raman Spectroscopy (SERS)

There are several clinical trials in progress with SERS. Sample types include blood [25,26], saliva [27], and tears. As reported in articles from two studies, SERS had a susceptibility of 80.7% and 84.1% in detecting squamous cell carcinoma of the oral cavity by analysing blood [28]. Therefore, the tests show that SERS biofluid is suitable as a sample and relies on metal nanoparticles for signal amplification. The status of clinical trials is important for the understanding and future prospect of SERS and Raman spectroscopy [29].

### 3.2. Coherent Anti-Stokes Raman Scattering (CARS) 

Conventional Raman spectroscopy uses only a CW laser to generate spectra, while CARS and SRS use two pulsed lasers with different wavelengths to enable nonlinear optical motions. CARS microscopy can form an optical contrast of endogenous chemical structures, which is popular in various fields of biomedicine as it can provide a high-resolution image. For example, CARS microscopy has been used to visualise tissue structures, skin [30], lung, kidney, and retina [31]. Consequently, CARS has been able to obtain micron-level images of brain slices, which has worked well in cancer diagnosis [32]. Concrete prostatectomy is considered the most popular method in civilised countries for curing members of the stronger sex with clinically localised prostate cancer. In this surgery, the entire prostate is removed, but the urinary ball is reunited with the urethra [33].

### 3.3. Resonance Raman Spectroscopy (RRS)

One of the drawbacks of Raman spectroscopy is the low signal intensity. This drawback can be corrected with RRS. By matching the wavelength of laser excitation to the electrical absorption maximum of a particular chemical, the Raman signal of certain bands is enhanced. The study was used for a multifaceted study of haemoglobin, and the release of cytochrome-c from mitochondria during apoptosis was also studied. Okada et al. [34] as well as other scientists have used RRS to perform unlabelled studies of molecular dynamics in apoptotic cells. Observation of mitochondrial membrane stained with the dye JC-1 using RRS confirmed that the observed release of cytochrome was due to apoptosis.

### 3.4. Spatially Offset Raman Spectroscopy (SORS)

Although SORS technology may not be approved in any way for uniform diagnosis of patients in our time, there are significant prospects for the eventual use of this technique in the clinic. Recent advances in medicine have shown SORS can be used in blood testing, such as assessing the quality of erythrocytes during blood transfusion in patients. Vardaki et al. [35] have shown that SORS is able to profile changes in oxygenation when stored for 6 weeks. It is well known that in blood transfusions, the chemical composition of blood units changes differently from time to time. For this reason, a few units over the years are by no means determinative of the relationship between erythrocytes properties. Feng et al. [36] used SORS to measure subcortical bone and biochemical changes with increasing depth in intact mouse bone.

## 4. Application of Raman Spectroscopy in Biomedical Research

**Figure 4:**
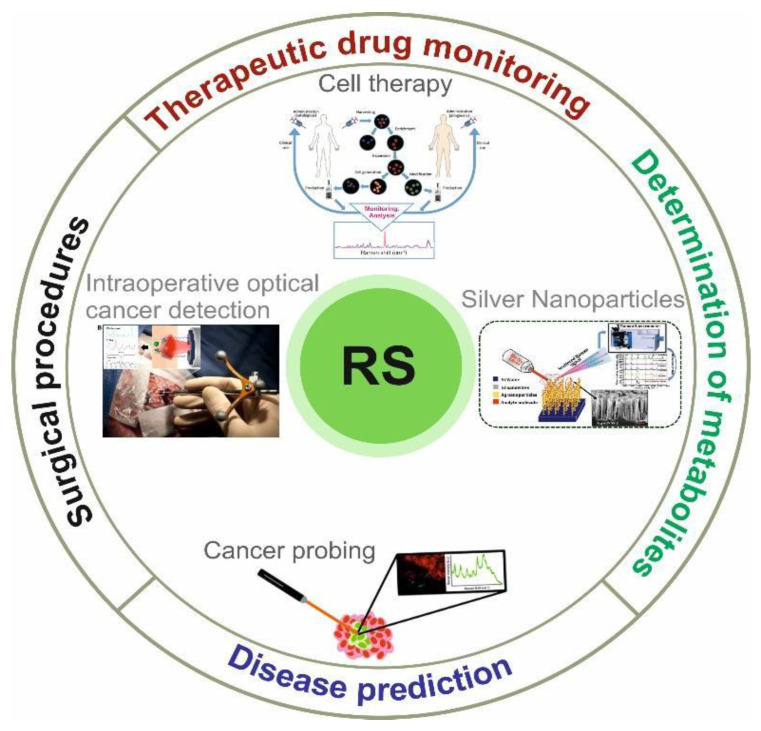
Schematic view of biomedical RS application. Adapted from Desroches et al. [37].

Due to the non-invasive, fast, and highly sensitive advantages of RS and its modifications, there is considerable demand for its use in biomedical research, such as in studying the structure and conformation of molecules of interest and investigating the mechanisms of the drug action [10,11]. Nowadays, RS is used in vivo and ex vivo to solve various biomedical issues, such as early cancer detection, monitoring the effects of different drugs on the skin, determining the composition of atherosclerotic plaques, and rapid identification of pathogenic microorganisms. Detailed information about the RS application can be found in Figure 4.

### 4.1. Disease Prediction

Early diagnosis of diseases, such as those that are life-threatening, is essential to prevent their spread. Based on Raman light scattering, a diagnostic tool has been developed to study important molecules and events in real-time using new RS technologies with high sensitivity to biomolecular changes. In the last decades, there have been many publications showing that RS is used to define several diseases [38]. RS is used to collect biochemical information, such as biomolecules, cells, tissues, and organs, whose biomarkers are various biological fluids, such as urine, saliva, blood, and tears. Selected biomarkers are analysed and evaluated, revealing the relationship between Raman light scattering spectral indicators and clinical condition [39].

Cancer is one of the most common causes of death worldwide and RS makes it possible to diagnose undetected precancerous lesions in various organs, such as breast, skin, brain, gastrointestinal tract, heart, urinary, and reproductive tracts [40]. Hsu et al. [41] investigated that confocal Raman microscopy distinguishes intestinal tumours from adenocarcinomas and normal, healthy organs. RS provides simple and immediate tissue identification during surgery, which allows for cancerous organs to be distinguished from healthy tissue. Using RS, the mechanism of malignant transformation of breast tissue has been studied with great success [42].

The properties of blood vessels in a tumour mass of breast tissue were investigated by Kopeć and Abramczyk [43] using a combination of Raman and atomic force microscopy (AFM) imaging to determine biochemical composition. They found that individuals with breast cancer had higher concentrations of glycogen and lactic acid as well as an increase in the collagen–fibroblast network. An excellent, recent study by Winnard et al. [44] demonstrated the potential of RS in characterising organ-specific metastatic lesions at the molecular level to gain insight into metastatic progression. In this study, they used the combinatorial approach of RS and metabolomics. The stromal adjustments that occur in pre-metastatic lungs caused by breast cancer were analysed using RS. This work was performed with mouse lines in which mice were implanted with breast cancer cells with different metastatic potential. Changes in the extracellular matrix of the congested lungs, such as an increase in collagen and proteoglycan, were examined, and this was directly related to the metastatic potential of the breast cancer cells used [45]. 

Ryzhikova et al. [46] have shown that RS can be used effectively for the diagnosis of Alzheimer’s disease (AD). The novelty of this work is that it is based on the analysis of cerebrospinal fluid (CSF), while the other research has focused on different body fluids for the detection of AD. It is important to emphasise that CSF is the most relevant body fluid for detecting AD. The group of Ryzhikova et al. [46] suggests that early detection of AD is potentially possible using RS. It is expected that the method will be repeated on a larger subject population.

Lednev group [47,48] diagnosed early AD in saliva and serum with potential biomarkers using RS in combination with machine learning. This project aimed to use Raman hyper-spectroscopy in combination with machine learning. New methods were developed to diagnose AD based on the analysis of biological material, such as saliva. The group used biological material from saliva samples from a normal person with AD and mild cognitive impairment. In the end, it turned out that Raman hyper-spectroscopic analysis of saliva could be effective for an accurate diagnostic method in the early stages of AD. It is also possible to diagnose lung cancer with high accuracy at an early stage, as shown by the studies of Shin et al. [49] using a combination of SERS spectra and deep learning diseases. The advantage of this method is that tissue can be seen in the near-infrared region of the electromagnetic spectrum, which in combination with the RS instrument as well as multivariate data analysis, has become an accurately reproducible and non-invasive method for studying tissue pathology.

Barnas et al. [50] used Fourier transform infrared (FTIR) and RS to study endometrial hyperplasia and cancer. The study was performed on tissues from three groups of patients: normal control patients, patients with atypical hyperplasia, and patients with endometrial cancer. It has been revealed that both methods are complementary in terms of tissue examination. The results of the research suggest that the peaks of FTIR and Raman spectra and the changes in the specific peaks (absence of the peak or shift of the peak) can be used to distinguish cancer and atypical hyperplasia from normal endometrial tissue. Further studies are needed to understand whether RS is indeed a practical approach to study carcinogenesis. 

SERS immunoassays have labelled/indirect or unlabelled configuration. Without labels, the Raman measurement is based on the fingerprint of the bioanalyte, and the labelled ones are identified by the spectrum of the Raman label. Therefore, labels without labels are not as complex as the labels that make up the labels on metallic nanostructures. Two systems were used to detect proteins, nucleotides, and fatty acids of lipids. The changes that occurred in the bioassay were recorded and diagnosed with infectious and non-infectious diseases [51]. Table 2 shows some examples of bioanalytes or diseases that were detected using SERS.

In addition, RS is used to analyse the serum of patients with AD, patients with other types of dementia, and individuals from the control group. The results were analysed using multivariate statistics for differential identification of patients with AD. The study was a confirmation of the concept; this proves that RS and artificial neural network classification were able to differentiate patients with sensitivity and specificity of more than 90%, which shows that a combination test can become a blood test that can support clinical evaluation for effective and accurate differential diagnosis of AD [62].

### 4.2. Surgical Procedures

In medicine, much attention is paid to optical instruments based on RS, which consist of intraoperative procedures in real-time. The benchmark for surgical guidance is histopathology, which also involves surgical removal of tissue followed by staining and examination under a microscope. This procedure takes a long time and in some cases, results in multiple biopsies, which causes a great deal of discomfort and suffering in patients [49]. Therefore, a sensory system that can provide results during surgery is needed. For example, endoscopic pain analysis before surgery, delineation of the sides of the lesion during surgery, and changes of the single biopsy using RS can contribute to the absolute removal of the affected tissue and reduce the cost of secondary assessments of the disease and surgery [63].

Motz et al. [64] have developed a small diameter Raman probe with integrated filters and a spherical lens to minimise low priority signals. In as little as one second, the probe can show the spectra of arteries and breast tissue at different stages of pathology, which is clinically useful. Jermyn et al. [65] studied cancers of multiple human organs during surgery with 97% accuracy using a trimodal optical imaging system combining Raman. Thus, the method demonstrated that molecular imaging with high sensitivity could dramatically impact such areas of surgical and non-invasive oncological procedures for tumour detection to reduce cancer risk and improve quality of life. Kircher et al. [66] investigated the ternary status of magnetic resonance imaging—photoacoustic Raman imaging of nanoparticles, which revealed brain tumour boundaries and visualised tumour margins using RS. They used Raman imaging to ensure monitoring of intraoperative tumour resection, and histologic interdependence proved that Raman imaging delineated brain tumour boundaries. This latest trimodal aspect using nanoparticles can ensure the clearest visualisation of even the resection of a brain tumour.

In addition, significant steps are being taken to integrate RS with other wide-field and spectroscopic methods to provide additional data to support RS measurements. It has been shown that cancer cells can be diagnosed in less than one second using the broadband fluorescence method together with a Raman micro-spectrometer [67]. The trimodal optical imaging system is a combination of Raman scattering, diffuse reflectance, and intrinsic fluorescence spectroscopy, in which various cancer organs were detected during surgery with 93%, 100%, and 97% accuracy [65]. Kircher et al. [66] also synthesised a nanoparticle with three-component magnetic resonance imaging—photoacoustic and Raman imaging with the aim of preoperative and intraoperative separation of the sides of the leading brain tumour; the presence of this RS was used to visualise the sides of the tumour.

Nowadays, RS is increasingly used for cancer diagnosis and monitoring. As mentioned above, the improvement of algorithms for processing Raman signals, as well as the development of new methods SERS and fibre-optic probes, may make it possible to obtain results with high sensitivity and specificity and to apply RS approaches to cancer diagnosis.

### 4.3. Therapeutic Drug Monitoring (TDM)

Therapeutic drug monitoring (TDM) is an important method in clinical pharmacology and clinical chemistry that aims to measure drug concentrations in human blood [54]. TDM has been used in medical practice since the 1960s and mainly focuses on drugs with narrow therapeutic targets [68,69,70].

TDM is more commonly referred to in clinical practice as the observation of drug concentrations in biological fluids over time [69,71]. Karine is important for drugs with a limited therapeutic index, where a low dose is prescribed when the difference in dosage may lead to serious therapeutic consequences such, as drug toxicity and side effects [72]. There is also increasing advocacy in the field of personalised medicine that will be useful for measuring the plasma concentration of a drug at individual doses. Individualised therapy planning in personalised medicine has become a great challenge for clinicians as it is very successful in improving patient care, so that each patient can reduce drug costs while receiving optimal treatment with minimal side effects. 

Fei et al. [73] performed the synchronous pharmacokinetics of 6-mercaptopurine and methimazole in the HeLa cell directions using the automated micro-fluid concept in the SERS database, which is convenient for synergistic tumour targeting. Valves and gradient generators can be used to adjust each of their chambers to deliver the required amount of different active cells and drugs at specific concentrations. The aforementioned examples also show that SERS is a robust method to determine the number of entrapped substances. 

Nowadays, there is a crucial problem of how to distinguish expired and non-expired drugs using a quick and non-invasive method. Current methods, such as HPLC, thin-layer chromatography, are time-consuming and complicated. In contrast, RS is a rapid and non-invasive method that can be used to examine expired and non-expired drugs. This is a significant problem for the medical world [72,74]. 

The combination of AFM and RS can distinguish the characteristics of the nucleus and cytoplasm in living cells. By combining both methods, a modification of RS can be developed: tip-enhanced Raman spectroscopy (TERS). Intracellular imaging with TERS has been applied to HeLa cells. It has been shown that the regions of the nucleus and cytoplasm can be effectively distinguished using this method, for which the local information within the cell was obtained. Crucially, scientists have shown that the viability of the cell membrane is very high (about 100%) after the AFM tip penetrates the cell membrane. The method has significant potential for future use in studies where it is necessary to investigate the various organelles and biomolecules within the cell [75]. 

### 4.4. Determination of Metabolites

Molecularly specific RS is well suited for profiling cellular metabolites, including neurotransmitters, amino acids, lipids, glucose, and nucleic acids, as well as in biofluids. Among all cellular metabolites, lipids are one of the most studied classes of biomolecules because they have large Raman scattering cross-sections. Lipids rich in intracellular bodies are referred to as lipid droplets, making their relationship to the physiological state of the cell increasingly apparent [76]. RS exploits the promise of detecting and imaging lipid droplets for quantification in cancer cells, such as HuH7 and colorectal cancer stem cells [77].

RS and its modifications are widely used in cell research. A variety of microalgae [6] and human [1,2,3] cells are studied using SERS. It has been shown that information about membrane lipids can be obtained, especially the conformation of membrane lipids and the molecular environment [1].

RS has been used to study nerve myelin during excitation or the effect of neurotransmitter on nerve fibre. It has been shown that the changes in the C–C bonds of the fatty acids can be detected as well as the changes in the conformation [78]. This knowledge will improve our understanding of the mechanisms of lipid–lipid interactions in myelin and many processes associated with various diseases, such as multiple sclerosis, trauma and AD.

It is important to add that human skin is exposed to ultraviolet and infrared radiation, which is the cause of a number of diseases and ageing of human skin. Carotenoids are considered antioxidants that can support the antioxidant status of the human epidermis [79]. RS is one of the most popular methods to study carotenoids. More knowledge about the mechanisms of the photoprotective function of carotenoids is important for biomedical research and the development of commercial products. Similarly, Gellermann et al. [80] used resonant Raman scattering spectroscopy (RRSS) as a novel, non-invasive, in vivo optical technique to measure the concentration of the carotenoid pigments, lutein and zeaxanthin, in the human retina of adolescents and adults. Using RRSS, they found an apparent decrease in macular pigment concentration during the normal ageing process. They suggested the use of RS to measure macular carotenoids as a promising technology. 

Ermakov et al. [81] reported that the RS technique has the potential for novel, rapid screening for carotenoid antioxidants in the largest populations at risk of vision loss due to age-related macular degeneration, an important precondition for blindness in the elderly in mature societies.

The method of RS is becoming increasingly popular in biological research. Valpapuram et al. [82] proposed a new technique combining optical biosensing and Raman micro spectroscopy. The particular advantage of this method is its ability to reduce the background signal and thus improve the signal-to-noise ratio. The researchers have shown that the combination of optical bio-sensing and Raman micro spectroscopy is a far more informative method than the conventional RS. 

SERS has been used to spatially localise neurotransmitters on living cells and to study protein–neurotransmitter interactions [83,84]. Although it offers the best detection limits, the toxicity of metal nanoparticles in vivo limits its use [85]. Manciu et al. [86] have also demonstrated the usefulness of confocal RS for rapid detection of neurotransmitter predictions, but their studies were limited by in vitro spiked material. They propose real-time detection of serotonin, adenosine, and dopamine in vitro, but in addition, diffusion of dopamine in a heterogeneous base gel used as a surrogate for neural tissue. Raman mapping was performed using alpha 300 WITec confocal Raman system to obtain non-overlapping spectral data of neurotransmitters. Their work demonstrates the power of Raman spectroscopy in the biological sciences and likely provides a novel mechanism for testing the adaptability and kinetics that stimulate the brain [86].

A rapid, non-invasive, label-free approach to biological studies is currently essential for scientific purposes. However, RS has some limitations—it requires longer acquisition times and it is not possible to optically slice the collected signal. This makes it difficult to use RS for tissue research alone. Therefore, Marchetti et al. [87] combined three methods: multiphoton microscopy, fluorescence lifetime imaging microscopy, and RS to perform an efficient study of tissues ex vivo. The mentioned tailored technique is a promising approach to expand the application of RS in biological research.

The use of the RS method in medical studies is becoming more common. For example, RS is used in dentistry. The short- and long-term effects of demineralization can be studied using the RS tool. The major advantage of RS is that it is non-invasive while providing a high degree of sensitivity. In the study of Marin et al. [88], quantitative information on the crystalline structure of the phosphate groups and the loss of the mineral fraction in the organic collagen matrix was discovered.

Nowadays, a precise, fast, and direct analysis tool is needed. The capillary sensor SERS, developed by the group of Arabi et al. [89], is proposed as an ultrasensitive tool and used for protein analysis. Trypsin is a protein that can be used as a biomarker (in urine) for the diagnosis of pancreatitis. The idea is that this approach can be effectively used for early diagnosis of the disease. In addition, and to test the feasibility of the tool, other biological fluids such as saliva and sweat have also been measured. The microsensors are relatively quick and inexpensive to produce. 

Another application of RS in biological studies is high-throughput screening Raman spectroscopy (HTS-RS)—presented in the work of Arend et al. [90]. This application is a customised platform for single-cell analysis. In the study in which the group of Arend et al. [90] examined the different types of neutrophils, both infected and uninfected, it has been shown that this type of platform can potentially help to speed up the diagnosis of pathogens. Currently, the routine for such analyses takes 1 working day. 

It has been discovered that RS can be used effectively in chronic renal failure (CRF) to differentiate patients with this disease from healthy patients. The group of Chen et al. [74] conducted a study on 47 samples from patients with CRF and 54 samples from control subjects. There is a prospect that the application used, which can be effectively utilised as a rapid diagnostic method for CRF. The plasma RS has been effectively used to study giant unilamellar vesicles (GUV)—simplified models of cellular plasma membranes [91]. The group of Collard et al. [91] has applied the modification of RS in combination with holographic optical tweezers (HOT): HOT-Raman microscopy for the study of curvature gradients on lipid order and cholesterol segregation in GUVs. The RS provides an overall estimate of cholesterol concentration for both leaflets of the bilayer. Importantly, the proposed method also allows for obtaining multiple Raman spectra from different regions of the lipid vesicle.

Raman spectroscopy is a promising high-sense diagnostic method for assessing the oxygen transport function of erythrocytes. Haemoglobin accounts for >95% of the dry weight of erythrocytes and is a suitable subject for RS to study the conformation of globin and haeme. To assess the conformational state of the active site of haemoglobin, we use special Raman spectra to study the conformation of deoxyhaemoglobin (d- Hb) and oxyhaemoglobin (o- Hb), as well as the ability to release oxygen. This approach is important for monitoring changes in the ability of haemoglobin in erythrocytes to carry oxygen and, accordingly, characterising the presence or development of hypoxia in patient tissues. RS has been successfully used to analyse the properties of haemoglobin from healthy donors and patients with various cardiovascular diseases [92,93], diabetes [94], and astronauts after a long space flight [95], as well as for the analysis of animal models of cerebral ischemia and reperfusion, haemorrhagic shock, etc. In addition, RS has been successfully used in experiments to alter the properties of erythrocytes under in vitro conditions. A promising application of RS is the study of the molecular mechanisms of the development of pulmonary hypertension. In patients with IPAH with a typical hemodynamic picture, changes in the ability of hematoporphyrin of Hb to bind O_2_ have been detected [96].

## 5. Biotechnology Application of Raman Spectroscopy

Biotechnology has become one of the most popular areas of research due to the demand for a number of molecules that are important for different types of practical applications [97]. Wang et al. provided a comprehensive and critical review of the most recent advances in the application of RS-enabled technologies, with focus on biomolecular applications in environmental and biotechnological fields [98].

Bioprospecting and mutagenesis are two important strategies that have been studied in the development of algae-based biofuels [18]. Considering these two strategies, there is a need to optimise biofuel production. The ability to rapidly characterise the accumulating algal lipids is essential for algal bioproduction. Confocal Raman microscopy can accomplish this task and it is also possible to localise lipid-rich regions within microalgal cells with high spatial resolution [18]. Among the existing methods, RS is the one that does not require additional long preparations of the research object.

It has been investigated that the chemical composition of lipid droplets can be obtained by using RS. It is claimed that the combination of CARS (coherent) and Raman micro-spectroscopy would allow accurate determination of the harvesting times for algae [4]. This is supposed to be one of the valuable interests in modern biotechnology.

It has been revealed that another modification of RS—single-cell Raman spectroscopy (SCRS) is applicable for gathering information about the lipid content of the cells and the degree of lipid unsaturation [22].

RS is one of the techniques that can fill some gaps in our knowledge of the synthesis and storage of biomolecules in algae [21].

### 5.1. Application of Raman Spectroscopy in Algae Studies

**Figure 5:**
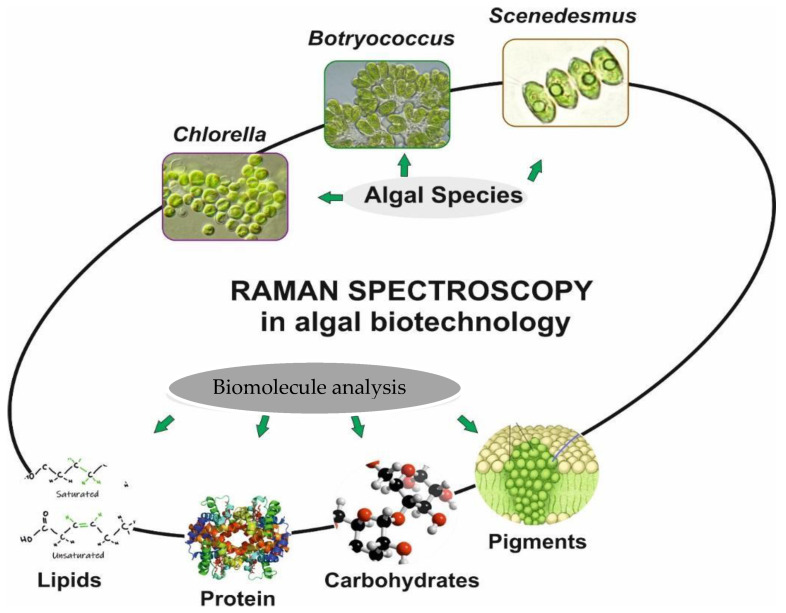
A schematic of the experimental set-up of a typical Raman spectrometer and schematic showing applicability of RS to different aspects of algae.

In algal research, RS has been used to analyse pigments, proteins, carbohydrates, and lipids [98,99,100,101,102] (Figure 5). Huang et al. [14] investigated the composition of microalgae to analyse them using confocal Raman microscopy. The scientists collected Raman spectra while using a 532 nm laser and found a strong background of fluorescence as a function of temporal behaviour. They also used RS to analyse the resolution of individual cells of microalgae. Kaczor et al. [103] published a study on the state of nutrients of single cells of microalgae and the visualisation of astaxanthin in a cell of microalgae, visualising Raman in situ with a 1064 nm laser. Laser capture Raman spectroscopy and a combination of laser capture and micro-Raman spectroscopy were developed by researchers Wu et al. [23] in which the lipid composition of microalgae was analysed using a single cell. For example, Hosokawa et al. [104] used the confocal Raman microscopy method to quantitatively monitor lipids based on a single cell. A brief report on the prospects for algal research based on RS was given in two published review articles [105,106]. Wei et al. [106] have reviewed some of the recent advances in RS applications in areas related to microalgae. Strategies based on RS provide tremendous potential for non-invasive biochemical-composition analysis and imaging of living microalgal cells. The analysis of lipids carried out by the ratiometric method has provided a solid basis, but to improve the quality of data collection and to obtain an accurate analysis, one hundred percent adjustment of the data collection parameters is required.

In addition, the bioactive components of many microalgae species have been studied using this Raman method. Micro-Raman spectroscopy is the most effective method for studying biologically active additives. The most commonly used modifications are macro-Raman spectrometry, single-cell micro-Raman spectrometry, and Surface-enhanced Raman Spectroscopy (Table 3).

As shown in Table 1, two species of microalgae were studied using single-cell micro- and macro- RS methods. *Arthrospira platensis* was the highest with 1118, 1403, and 1576 cm^−1^. The significant changes (1118, 1403, and 1576 cm^−1^) are explained by considerable differences in the structure of secondary proteins, especially amide bonds (1400 cm^−1^) and the α-helix (1574 cm^−1^) [113,114]. Furthermore, Venkatesan et al. [115] indicated that the wavenumbers of *Arthrospira platensis* (1030–1120 cm^−1^) are responsible for the presence of antioxidant protein enzymes. *Phaeodactylum tricornutum* was found to have high wavenumbers of 1522 and 1160 cm^−1^. Moudříková et al. [116] found that 1160 cm^−1^ corresponds to the polyphosphate-positive groups of microalgae. The authors used RS for quantification as well as for localisation of polyphosphate reserves within an algal cell. These authors improved the method by extracting polyphosphate with phenol–chloroform and by purifying the extract through ethanol precipitation. Wei et al. [106] noted that they (1518–1525 cm^−1^) correspond to the C–C regions of β-carotene, which are mainly present in the non-polar phase of microalgae. Brahma et al. [108] observed the strongest peaks around 1527 and 1158 cm^−1^ in *Dunaliella tertiolecta* associated with carotenoid pigments. Huang et al. [14] studied *Chlorella sorokiniana* and *Neochloris oleoabundans*. *Chlorella sorokiniana* had strong peaks at 2800 and 3000 cm^−1^. Further support for the use of a broad band of wavenumbers for lipid identification is provided by several previous CARS studies that used peak positions at 2840 or 2845 cm^−1^ for lipid identification [106]. Osterrothová et al. [20] tested the possibilities of Raman micro-spectroscopy for the determination of carotenoid pigments, both primary (lutein, β-carotene) and secondary (astaxanthin) carotenoids. The components of the xanthophyll cycle, violaxanthin (1525 cm^−1^) and antheraxanthin (1523 cm^−1^), can also contribute to a shift in the position of the ν1 (C=C) band. Strong carotenoid signals were observed by Jehlička et al. [117] in *Botrydiopsis alpina* at 1527 cm^−1^. In *Dunaliella parva*, the secondary carotenoid neoxanthin was found at 1525 and 1530 cm^−1^.

*B. brauniii* is a classic producer of liquid hydrocarbons known as botryococci, which can be used as fuel. Weiss et al. [111] have obtained Raman spectra of hydrocarbons isolated from *B. brauniicells*. By comparison with the Raman spectra of pure squalene and computer analysis, they found that the Raman bands at 1640 cm^−1^ and 1647 cm^−1^ were related to the stretching of the C=C bond in the botryococcus branch and the methylene C=C bond. produced by methylation, respectively.

Compared with the standard fatty acid mixture, the main SERS peaks obtained from the cells of *S. quadricauda* are at 1430, 1157, 1544, 1257, 1307, 961, and 596 cm^−1^, which is in good agreement with the literature data [22,112,118].

#### 5.1.1. Raman Spectroscopy Applied to Lipid

Normally, lipid droplets are assumed to be cellular structures that function only as static lipid storage depots. Recently, however, it has become apparent that lipid droplets may be multifunctional organelles [119,120]. Considering the information available so far, there is a need for deeper research into lipid droplets—their structures and functions in cells. Nowadays, there is a hypothesis that lipid droplets may be involved in the biosynthesis and transport of carotenoids in the cell [121]. It is known that the biosynthesis of the carotenoid astaxanthin is accompanied by a massive accumulation of lipids [122].

Another interesting finding is that the membrane fluidity of cyanobacteria can change when the temperature is lowered [123]. This is thought to be due to the desaturation of fatty acids in the membranes [124]. RS is an attractive alternative for lipid detection that has not yet been sufficiently exploited in microalgae. Mostly this is because RS is hampered in photosynthetic organisms by strong autofluorescence of the pigments, which obscures the characteristic Raman spectral features.

The intensities of the Raman spectral peaks correspond to the saturated and unsaturated C–C bonds in lipid molecules. This information is used to estimate the degree of unsaturation in lipid bodies/droplets [4,5,15,16,18]. It has been shown that Raman micro spectrometry can be used to study the triacylglycerols (TAG) content and accumulation [21], providing a new view of the biosynthesis of fatty acids in the microalgae.

**Figure 6:**
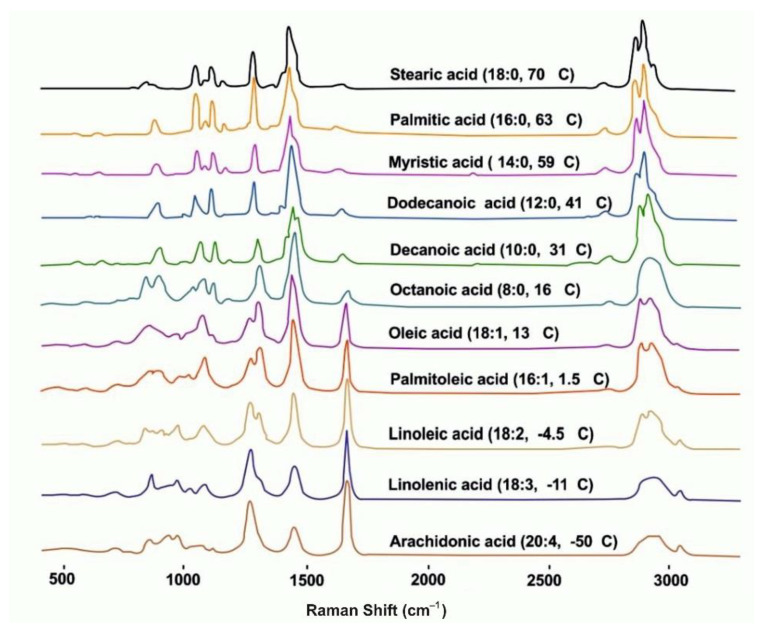
Raman spectra of various lipid molecules of *Botryococcus braunii* [23].

Several authors have shown that the RM methods are suitable for biodiesel production from microalgae to determine the FAs content. Wu et al. [23] demonstrated a method for direct quantitative and in vivo lipid profiling of oil-producing microalgae using single-cell RS laser capture. This approach shows that lipids in microalgae determine the quantitative degree of unsaturation and the transition temperature. As the authors stated, the above factors can be measured on a single living cell of a microalga held in place with an optical trap while Raman data is collected. Raman is used in the study of FAs from microalgae for biofuel production and has shown analytical capabilities and quantification algorithms to be useful in many different organisms and lipidomics (Figure 6).

Another ability of Raman microscopy reported by Samek et al. [5] showed how the useful iodine number in lipid bodies in *Chlamydomonas* sp. CCALA can be determined from living algal cells. At the same time, the characteristic peaks in the Raman light spectra at 1656 cm^−1^ and 1445 cm^−1^ were used as markers for fatty acids in algae lipids, indicating the ratio of unsaturated and saturated carbon-carbon bonds [5].

He et al. [19] investigated the accumulation of separation of TAG in *Coccomyxa subellipsoidea* cells in the presence of N-depletion using the broadband CARS concept. Compared to simple Raman imaging, CARS microscopy showed intrinsic advantages in detection speed and spatial resolution, but the concept of CARS imaging was limited by overlapping signals, such as two-photon-excited fluorescence.

Yan et al. reviewed several RS methods in cell sorting to understand the metabolic interactions between bacteria in natural habitat. This review shows current knowledge about the research progress of recognition and assessment of single microorganism cell. The group summarised that Raman-activated cell sorting can be suitable method for cell recognition in application [125].

**Figure 7:**
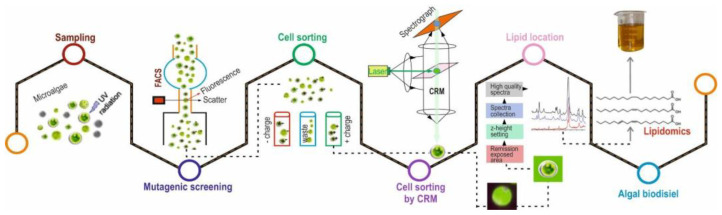
The schematic view in lipid characterisation of microalgae. Bioprospecting of *C. reinhardtii* is performed to generate algal samples with lipid content. The mutagens are sorted by FACS based on the fluorescence of a dye to select cells with high lipid content. The selected cells and mutants are then screened using CRM. This method allows for rapid characterisation of lipids. The spectra yield depends on the number of C=C bonds and the length of the hydrocarbon chains of the lipid molecules. This workflow enables rapid characterisation of cells for molecular traits that are important for the production of biodiesel. Modified from Sharma et al [18].

Thus, Raman spectrometer is used in microalgal biotechnology to screen species for highly concentrated fatty acid mutants [18]. There are two major strategies in algal biofuels development: bioprospecting and mutagenesis. This requires precise sorting and analysis of a large number of algal isolates containing TAG; FACS and ratiometric Raman analysis are the most suitable. Isolation of new algae from field samples can be carried out by UV mutagenesis to increase lipid production. FACS can be used to sample mutant populations and strains that alter lipid production during UV mutagenesis. Central to this workflow is confocal Raman microscopy, which allows for characterisation of the lipids produced by the algae in situ and rapid extraction of lipids from the cells. Raman hyper-spectroscopy is used to localise the lipid-rich region with a low pixel density, allowing faster Raman hyper-spectroscopy imaging. Confocal Raman microscopy characterises the lipid content (Figure 7).

#### 5.1.2. Application of Raman Spectroscopy on Pigment Investigation in Microalgae

Thus, considering the spectra of the pigments, they are very sensitive to the excitation energy and contribute to a large extent to the Raman spectra of many algae [108,125,126,127,128]. Chen et al. showed that when a long excitation wavelength of 488 nm is used, the strongest and most abundant peaks of chlorophyll-d coincide with the peaks of chlorophyll a and chlorophyll b [129]. β-Carotene has intense peaks at 1150 cm^−1^, 1520 cm^−1^, and 1008 cm^−1^, and the most important overtone peaks at 2320 cm^−1^ and 2667 cm^−1^ [130,131,132,133]. In addition, due to the identical chemical structure, it is expected that a large number of compounds of both chlorophyll and carotenoids will have similar spectra.

**Figure 8:**
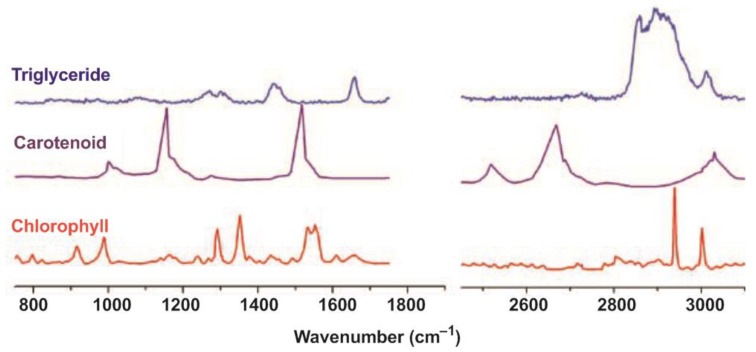
The Raman spectrum of carotenoid [132], chlorophyll [129], and triglyceride and the mean spectra acquired for starved *C. sorokiniana* and starved *N. oleoabundans* in the wavenumber regions of 750–1750 cm^−1^ and 2450–3150 cm^−1^. Modified from Shutova et al. [134].

Furthermore, because of the similar chemical structure, we would expect different chlorophyll compounds to give similar spectra and different carotenoids to give similar spectra. Therefore, the major Raman peaks associated with chlorophyll d and β-carotene can be used to represent common chlorophylls and carotenoids. The standard spectra of chlorophyll d and β-carotene are shown together with the experimental spectra of algae (Figure 8).

Several researchers have shown that RS methods are also suitable for the production of carotenoids by microalgae. Carotenoids are extremely important for human health [135]. Carotenoids are popular biomolecules for biomedical applications. Carotenoids are known to play the role of photoprotection molecules in the cells of phototrophs. Secondary carotenoids are also of interest as secondary carotenogenesis is thought to be the stress response of the cell [121]. 

It is also believed that carotenogenic microalgae can survive in a wide range of environmental conditions [136]. Nowadays, the analysis of carotenoids in algae is performed using high-performance liquid chromatography (HPLC). However, it is important to emphasise that RS can be used for a more detailed analysis of carotenoids in algal cells [17,20,24]. In addition, the development of RS, which will be applied to algae in the future, can be used for real-time research of algal combination in nature.

Jehlička et al. [7] studied how RS can be used to identify various carotenoids as well as probable biomarkers in algae. A number of laboratory grown algae with different taxonomic groups were studied. The results showed that RS is considered an optimal tool for assessing the presence of carotenoids in a given organism. The comparison was made with the HPLC method to examine the pigments in the concentrates. In summary Raman spectroscopy can be used for the detection of carotenoids and other pigments in algae.

Osterrothová et al. [20] tested the abilities of RS to determine carotenoid pigments—both basic (lutein, β-carotene) and secondary (astaxanthin) carotenoids—in different species of *Chlamydomonadales* algae. They also compared the performance of RS with a standard biological pigment analysis method, such as HPLC. They described the carotenoids of algae using a combination of resonance RS and HPLC, also creating a spectral library for different stages of the algal life cycle. A comprehensive study to find pigments in biomass can show results with HPLC. However, this method requires the extraction of pigments from the biomass, which can lead to data loss (e.g., protein/lipid interactions). Raman macro–microscopy, however, makes it possible to quickly reveal the pigments of single cells, which is another advantage, especially when the heterogeneous nature of the cells is taken into account.

To map the changes in the composition of β-carotene and AXT in different cellular morphotypes of *H. pluvialis*, Collins et al. [137] used a confocal Raman microscope at 532 nm laser excitation. Using a multivariate curve, several readable spectral components were extracted from the data describing Raman scattering and fluorescence of active *H. pluvialis* cells at different life stages. Based on the results, they were able to determine the arrangement of the different pigments in the cells at different time periods. They also concluded that β-carotene can be considered as an ancestor of AXT and a site for the synthesis of AXT. Their study shows that Raman micro-spectroscopy is an important method for studying in vivo changes stimulated by the environment in the life cycle of microalgae.

In their study, Chiu et al. [138] demonstrated for the first time that RS can be used to quantify starch in addition to lipids in algal cells. Because RS is so simple and non-destructive, it is ideal for further investigation of the starch–lipid shift mechanism.

RS provides information about the vibrations of bonds in molecules. This approach is used for the study of carotenoids. It has been shown that changes in the molecular environment (such as pH change) affect the specific bands (Figure 8) in the carotenoids’ spectra [134].

This is particularly attractive for applied sciences, such as biotechnology and biomedicine.

## 6. Raman Spectroscopy for Photosynthetic Studies

Photosynthesis is the most basic and important process on earth. It is the natural way of synthesising carbohydrates using solar energy. Scientists from all over the world are exploring it with a number of applications, one of which is Raman spectroscopy. Findings from a number of recent studies on RS applied to photosynthetic organisms are shared below. 

Mishra et al. [139] recently conducted their study on Antarctic lichens using RS. Antarctic lichens are organisms that can change their metabolism and photosynthetic activity in response to changing environmental conditions. Hydration and dehydration are the investigated triggers for the activation/deactivation of photosynthetic processes in the lichens. It has been revealed that photosynthetic activity is activated quite rapidly, which contributes to the hypothesis that the photosynthetic apparatus and carotenoids are not synthesised de novo in the early stages of photosynthesis. Another important discovery made using RS, the bands/features of the pigment scytonemin, are present in the Raman spectra of one of the lichens studied. There is a hypothesis that this pigment plays a photoprotective role in the photobionts of algae and cyanobacteriae.

## 7. Raman Spectroscopy for Analytical Studies

There is an ongoing need for fast and accurate detection of melamine in dairy products. Melamine is a compound that can be toxic above a certain level when added to food. Therefore, it is important to propose an approach that allows accurate detection of the toxic compound in food products. Liu et al. [120] proposed the SERS method using silver nanoparticles (AgNPs). Not only SERS but also a colourimetric method was used for this idea. The results showed that the colourimetric method can lead to false-positives in detecting the presence of different compounds (AgNPs). The SERS method, on the other hand, can overcome this limitation [120]. Importantly, the scientists suggested using both methods in tandem to achieve accurate and rapid detection of melamine in dairy products.

Fentanyl is one of the most commonly used opioids. However, fentanyl and its analogues caused numerous fatal drug overdose incidents. The problem raised by the group of Mirsafavi et al. [140] is the need for novel analytical methods to effectively distinguish fentanyl from its precursors. The vibrational spectra of this family of analytes are quite similar, so it is difficult to solve the problem using conventional methods. The SERS method enables the distinguishing of fentanyl and its precursors. This approach would be an efficient and effective aid in the field of forensics.

## 8. Future Perspectives

In recent decades, RS has successfully emerged as a clinical tool for diagnostic, surgical, and pathological applications. The creation of probes in conjunction with modern methods of studying information has led to a surge in studies based on combinatorial light scattering. However, when introducing RS into clinics, there are the major difficulties described in the previous section, which should be overcome by close collaboration between clinicians, material scientists, biomedical engineers, and spectroscopists. Artificial intelligence algorithms are expected to be used to solve complex clinical issues, which will accelerate the work of RS. In addition, the probes must be resistant to disinfection for daily use. Further advances in scientific and technical research, which also guarantee a high signal-to-noise ratio with the lowest laser excitation power in a short time, are accordingly worth examining in order to use RS for intraoperative procedures. Nevertheless, introducing new technology into the clinic remains a challenge, even though recent successes and prospects represent meaningful ideas for us and inspire us to solve certain complex problems, opening the door to an appreciable goal.

In addition, Raman spectroscopy is a common tool for detecting carotenoids in various biological materials, including prokaryotic bacteria, aquatic plants, and lichens. The resonant Raman amplification of the signals enables the detection of carotenoids at low concentrations. In other cases, however, microorganisms also synthesise other pigments, and the examples studied included their composition. In this case, the combinatorial scattering ranges do not at all include a series of sudden bands corresponding to this carotene, nor was there any significant broadening of the bands. The predominant carotenoid can be seen in the spectra. However, it was not possible to use a unique excitation wavelength from a range of microorganisms.

Raman spectroscopy can be used to detect the presence of carotenoids and other pigments in cyanobacteria, microorganisms, and algae. The occurrence of colour combinations in this organism is capable of producing small or moderately significant changes in saturation and in the number of combinatorial scatter bands, which interferes with the likely unambiguous identification of carotenoid c due to shifts in power, particularly in the position of the combinatorial scatter bands.

In recent years, RS has been widely used in research, including photosynthesis and analytical research. However, very little research has been conducted on plant photosynthesis using RS. Most research has been carried out to determine the composition of pigments in cyanobacteria.

## 9. Conclusions

RS and a spectrum of different modifications of the RS method are increasingly used in biological and medical research. RS is gradually becoming more popular among algae experts. In the list of studies, it has been revealed that we can successfully detect and analyse the Raman scattering signal of algae. This is important not only for biotechnology, but also for a better understanding of the mechanisms of the biomolecule synthesis and storage in algal cells. In this review, we have analysed and presented a number of existing studies in biological, medical, analytical, photosynthetic, and algal research using RS. RS is effectively and widely used for a variety of studies in animals and human research. We have attempted to highlight that a greater focus on the application of RS in algal research will be beneficial for biotechnological purposes and general knowledge of the mechanisms of the biomolecule interactions in algae under natural/environmental conditions. It is worth emphasising that RS is a very attractive and promising approach for algal research, especially because of its advantages.

## Figures and Tables

**Table 1:** The list of modifications of RS with details of the objects and molecules of interest with reference numbers of the papers used in the article.

Modification of Method	Object	Biomolecules	Reference/Link Number
Coherent anti-Stokes Raman scattering (CARS) and microscopy	microalgae	lipids, carotenoids	[4,15,16,17]
Confocal Raman microscopy	microalgae, algae	lipids	[18,19]
Raman micro spectroscopy	algae, animals	lipids, carotenoids	[2,5,20]
Resonance Raman spectroscopy (RRS)	bacteria, microalgae	carotenoids	[7,19,21]
Single-cell Raman spectroscopy (SCRS)	microalgae	lipids	[22,23]
Surface-enhanced Raman spectroscopy (SERS)	animals, bacteria, microalgae	lipids, carotenoids, proteins	[1,2,6,10,14,24]

**Table 2:** Bioanalytes/diseases detected using SERS.

Bioanalyte/Disease	RS Substrate	Reference
Cancer (blood plasma protein)	Ag NPs	[51]
Quantification of hepatitis B DNA	Ag NPs	[52]
Breast cancer tissue	Ag NPs	[53]
Sjogren’s syndrome from saliva	Cl-Ag NPs	[54]
Human tear uric acid	SiO_2_ and Au	[55]
Creatinine	Nano-Au	[56]
Mouse IgG	Au NPs	[57]
Single prostate cancer cells	Au NPs	[58]
Plasmodium falciparum DNA	Magnetic beads	[59]
HeLa cells	Au NPs	[60]
Gastritis	Au NPs	[61]

**Table 3:** Summary of the bands observed in the Raman spectra of microalgae and contributing bioactive compounds.

Bioactive Compounds	Microalgal Strain Name	Type of RS	Wavenumber	Ref.
α-helix protein	*Arthrospira platensis*	Macro-Raman spectrometry	1574 cm^−1^	[107]
Amide bonds	*Arthrospira platensis*	Macro-Raman spectrometry	1400 cm^−1^	[107]
Antioxidant protein enzyme	*Arthrospira platensis*	Macro-Raman spectrometry	1030 and 1120 cm^−1^	[107]
Polyphosphates	*Phaeodactylum tricornutum*	Single-cell micro-Raman spectrometry	1160 cm^−1^	[107]
ß-carotene	*Phaeodactylum tricornutum*	Single-cell micro-Raman spectrometry	1522 cm^−1^	[107]
ß-carotene	*Dunaliella tertiolecta*	Resonance Raman spectrometry	1158 and 1527 cm^−1^	[108]
Triglyceride	*Chlorella sorokiniana*	Micro-Raman spectroscopy	2800 and 3000 cm^−1^	[14]
ß-carotenoid	*Neochloris oleoabundans*	Micro-Raman spectroscopy	1505 and 1535 cm^−1^	
ß-carotene	*Chlorella sorokiniana*	Raman micro-spectroscopy	1515 and 1157 cm^−1^	[109]
Astaxanthin	*Chlainomonas* sp.	Micro-Raman spectroscopy	1520 and 1156 cm^−1^	[20]
Astaxanthin	*Chlamydomonas nivalis*	Micro-Raman spectroscopy	1520 and 1156 cm^−1^	
Violaxanthin	*Chloromonas nivalis*	Micro-Raman spectroscopy	1525 cm^−1^	
Antheraxanthin	*Chloromonas nivalis*	Micro-Raman spectroscopy	1523 cm^−1^	
Myxoxanthophyll	*Botrydiopsis alpina*	Micro-Raman spectroscopy	1527 cm^−1^	[110]
Neoxanthin	*Dunaliella parva*	Micro-Raman spectroscopy	1525 and 1530 cm^−1^	
Chlorophyll c	*Dunaliella tertiolecta*	Micro-Raman spectroscopy	1670 cm^−1^	
Lipid	*Botryococcus brauniiis*	Micro-Raman spectroscopy	1640 and 1674 cm^−1^	[111]
FAME	*Scenedemus quadricauda*	Surface-enhanced Raman spectroscopy	1430, 1157, 1544, 1257, 1307, 961 and 596 cm^−1^	[112]

## Data Availability

The data presented in this study are available on request from the corresponding authors. The data are not public.
